# Diaphragm thickening in cardiac surgery: a perioperative prospective ultrasound study

**DOI:** 10.1186/s13613-019-0521-z

**Published:** 2019-04-24

**Authors:** Pierre-Henri Moury, Adrien Cuisinier, Michel Durand, Jean-Luc Bosson, Olivier Chavanon, Jean-François Payen, Samir Jaber, Pierre Albaladejo

**Affiliations:** 1 Department of Anesthesia and Intensive Care Medicine, Université Grenoble-Alpes, Grenoble University Hospital, Grenoble, France; 2 Department of Biostatistics, ThEMAS, TIMC, UMR, CNRS 5525, Université Grenoble Alpes, Grenoble University Hospital, Grenoble, France; 3 Department of Cardiac Surgery, Université Grenoble Alpes, Grenoble University Hospital, Grenoble, France; 4 Intensive Care Unit, Anesthesiology and Critical Care Department B, Saint Eloi Teaching Hospital, Université Montpellier 1, Centre Hospitalier Universitaire Montpellier, Montpellier, France; 5 Department of Anesthesia and Intensive Care, ThEMAS, TIMC, UMR, CNRS 5525, Université Grenoble-Alpes, Grenoble University Hospital, Grenoble, France

**Keywords:** Weaning, Mechanical ventilation, Cardiac surgery, Muscle

## Abstract

**Background:**

Diaphragm paresis is common after cardiac surgery and may delay the weaning from the ventilator. Our objective was to evaluate diaphragm thickening during weaning and secondly the muscle thickness as a marker of myotrauma.

**Methods:**

Patients undergoing elective cardiac surgery were prospectively included. Ultrasonic index of right hemidiaphragm thickening fraction (TF) was measured as a surrogate criterion of work of breathing. A TF < 20% was defined as a low diaphragm thickening. Measurements of TF were performed during three periods to study diaphragm thickening evolution defined by the difference between two consecutive time line point: preoperative (*D* − 1), during a spontaneous breathing trial (SBT) in the intensive care unit and postoperative (*D* + 1). We studied three patterns of diaphragm thickness at end expiration evolution from *D* − 1 to *D* + 1: > 10% decrease, stability and > 10% increase. Demographical data, length of surgery, type of surgery, ICU length of stay (LOS) and extubation failure were collected.

**Results:**

Of the 100 consecutively included patients, 75 patients had a low diaphragm thickening during SBT. Compared to TF values at *D* − 1 (36% ± 18), TF was reduced during SBT (17% ± 14) and *D* + 1 (12% ± 11) (*P *< 0.0001). Thickness and TF did not change according to the type of surgery or cooling method. TF at SBT was correlated to the length of surgery (both *r* = − 0.4; *P* < 0.0001). Diaphragm thickness as continuous variable did not change over time. Twenty-eight patients (42%) had a > 10% decrease thickness, 19 patients (29%) stability and 19 patients (28%) in > 10% increase, and this thickness evolution pattern was associated with: a longer LOS 3 days [2–5] versus 2 days [2–4] and 2 days [2], respectively (ANOVA *P *= 0.046), and diaphragm thickening evolution (ANOVA *P *= 0.02). Two patients experience extubation failure.

**Conclusion:**

These findings indicate that diaphragm thickening is frequently decreased after elective cardiac surgery without impact on respiratory outcome, whereas an altered thickness pattern was associated with a longer length of stay in the ICU. Contractile activity influenced thickness evolution.

*Trial registry number* ClinicalTrial.gov ID NCT02208479

**Electronic supplementary material:**

The online version of this article (10.1186/s13613-019-0521-z) contains supplementary material, which is available to authorized users.

## Background

Respiratory complications are frequent after cardiac surgery, and they have been related to diaphragm paresis [1–3]. Diaphragm paresis incidence depends mainly on surgical technique and is associated with delayed weaning from mechanical ventilation [4, 5]. It is classically related to phrenic nerve injury caused by hypothermia or mechanical trauma [6]. However, diaphragm paresis observed in cardiac surgery may also share the same pathways involved in ventilator-induced diaphragmatic dysfunction [7, 8]. It is caused by diaphragm disuse during mechanical ventilation or in sepsis as described in ICU settings [9]. Diaphragm disuse may be frequent after cardiac surgery because of ventilator settings under cardiac bypass and mechanical ventilation. Incidence of ventilator-induced diaphragm dysfunction may explain the frequency of respiratory muscle impairment in difficult to wean patient after cardiac surgery [10]. Assisted mechanical ventilation is used during weaning and the matter is the balance between low assistance leading to atrophy with over assistance generating asynchrony [11, 12]. In times of personalized physiological medicine, diaphragm myotrauma is an interesting model of the crosstalk between treatment monitoring and organ dysfunction [13]. This highlights the need for a direct measurement of diaphragm effort at the early phase after surgical injury [10].

Ultrasonic assessment of diaphragm thickening has been described as a noninvasive and reproducible method at bedside [14–16]. Assessment of the diaphragm thickness in the zone of apposition during the respiratory cycle provides specific information on the muscle strength [14, 15, 17]. The diaphragm thickening fraction (TF) has been proposed as a surrogate criterion to estimate the breathing workload [16, 18], and was found as a predictor of weaning success in the ICU settings [19]. It has been recently shown to be correlated with diaphragm strength, independently of peripheral muscular strength [20, 21]. Growing evidence exists that changes of diaphragm thickness might be related to diaphragm activity under assisted mechanical ventilation. Therefore, maintaining an appropriate level of inspiratory diaphragm contractility may play a key role in a muscle-protective strategy [22].

The primary objective was to assess diaphragm thickening measured by ultrasound at bedside during weaning from mechanical ventilation after elective cardiac surgery. We hypothesized that diaphragm thickening was frequently low after cardiac surgery.

## Patients and methods

### Study design

This physiological prospective study approved on June 12, 2014 (CECIC Rhône-Alpes-Auvergne, Clermont-Ferrand, IRB 5891), and registered at ClinicalTrial.gov (ID NCT02208479) was conducted from July to November 2014. Since this study did not modify current strategies, the need for written consent was waived according to French Law (Law 88-1138 relative to Biomedical Research of December 20, 1988, modified on August 9, 2004). Oral consent was obtained from each patient.

### Study population and procedure

Patients were prospectively and consecutively included in the study if they were hospitalized for elective cardiac surgery with cardiopulmonary bypass (CPB). Non-inclusion criteria included non-elective surgery or ICU stay before the procedure.

### Surgical procedure and perioperative patient management

Mammary, gastroepiploic and radial arteries and saphenous veins were used as coronary bypass grafts. Myocardial protection was provided using cold crystalloids and/or cold blood cardioplegic solutions. Different topical hypothermia methods were used including ice gauze dressings, cold saline or intra-pericardial slushed ice. According to surgeons’ preference, patients received either a crystalloid cardioplegia (modified St Thomas solution, Plegisol, Pfizer France, Paris) or a blood cardioplegia composed of a mix of one part of blood and four parts of crystalloid (Plegisol). Both cardioplegic solutions were infused with a temperature of 4–8 °C with a roller pump (Stockert Instrumente GmbH, Munich, Germany). Only anterograde administration was used for the crystalloid cardioplegia, the blood cardioplegia was administered both in an anterograde and retrograde manner through the coronary sinus.

During surgery (except cross-clamping period), mechanical ventilation was set at a tidal volume of 6–8 ml/kg of predicted body weight and a positive end-expiratory pressure (PEEP) level of 5 cmH_2_O, respiratory rate was adjusted to maintain normocapnia, and the inspired fraction of oxygen (FiO2) was set to keep PaO2 below 120 mmHg. On admission to the ICU, mechanical ventilation was adjusted with a PEEP level of 7 cmH_2_O. The absence of bleeding, normothermia and hemodynamic stability was checked prior to stopping sedatives and performing a spontaneous breathing trial (SBT). The ventilator settings were as follows: pressure support ventilation (PSV) of 7 cmH_2_O with a PEEP level of 7 cmH_2_O in a 30° upright position. Patient’s consciousness and the absence of neurological impairment were assessed during the SBT before release from mechanical ventilation.

On the day of surgery, anesthesia was induced and maintained using a target-controlled infusion of propofol and remifentanil. A neuromuscular blocking agent (cisatracurium, 0.12 mg/kg) was used to ease tracheal intubation and before rewarming without reinjection or reversal. Antibiotics (cefazoline) were administered before the incision and during CPB.

### Study protocol

**Fig. 1:**
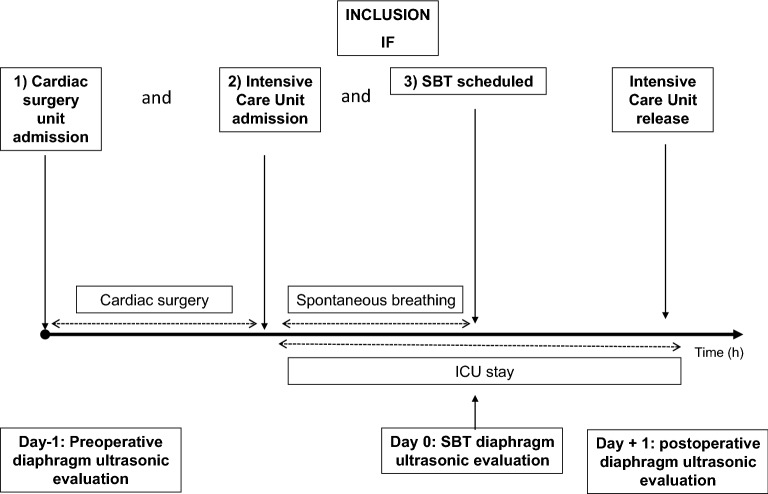
Study protocol. Each patient hospitalized in cardiac surgery with elective cardiac surgery and ICU hospitalization was consecutively included when the diaphragm thickening fraction (TF) could be measured during spontaneous breathing trial (SBT). Preoperative (Day − 1) and postoperative (Day + 1) were measured when feasible to assess perioperative evolution. Diaphragm function was evaluated by the TF measured on the right hemidiaphragm as reference in the same semi-recumbent position. SBT: Spontaneous Breathing Trial; ICU: Intensive Care Unit; TF: diaphragm thickening fraction

The study protocol is described in Fig. 1. Demographic data, severity scores, comorbidities, surgical and anesthetic data were prospectively recorded. Patients were included when diaphragm assessment of the right hemidiaphragm during SBT was feasible (primary outcome). In addition and if feasible, left hemidiaphragm was also assessed during SBT (secondary outcome reported in Additional file 1).

In order to study the time course of diaphragm thickening, assessment was performed on the day before (*D* − 1), and on postoperative day 1 (*D* + 1).

Respiratory variables and blood gas analysis were noted during SBT. Total length of mechanical ventilation, duration of ICU stay, extubation failure and ICU mortality were also collected. Extubation failure was defined as the need for reintubation within 48 h of extubation.

Spontaneous breathing trial (SBT) ventilator settings were as follows: pressure support ventilation (PSV) of 7 cmH_2_O with a PEEP level of 7 cmH_2_O in a 30° upright position [23]. Diaphragm thickening was assessed using ultrasounds 30 min after the initiation of SBT, on the right hemidiaphragm, by setting the PSV at 0 cmH_2_O and PEEP level fixed at 0 cmH_2_O. The ultrasound was conducted within 10 min after the initiation of this ultrasound setting to reflect function under stress condition rather than resistance to fatigue [24]. These settings were used to avoid a PEEP recruitment effect on diaphragm position in order to enhance the sensitivity of the exam by lowering the pressure support and to reproduce post-extubation respiratory physiology values [8, 25]. Diaphragm thickening was evaluated during SBT because this timing is relevant to evaluate post-extubation effort, weaning failure and the impact on outcome. [21, 25–27].

Ultrasounds measurements were recorded (DICOM viewer 3.0, Philips, Netherlands) and averaged over at least three respiratory cycles. Diaphragm thickness was measured in the zone of apposition of the diaphragm to the rib cage between the 8th–10th intercostal spaces using a 7.5–10 MHz probe [14, 28]. Measurements were done after careful observation of real-time graphics of airflow and airway pressure, at the end of inspiration (*T*_EI_, mm) and expiration (*T*_EE_, mm). *T*_EI_ and *T*_EE_ were measured to calculate the diaphragm thickening fraction (TF) as (*T*_EI_ − *T*_EE_)/*T*_EE_ and expressed as percentage. To study diaphragm *T*_EE_ evolution, we divided a posteriori the study population into three groups based on the change in diaphragm *T*_EE_ from *D* − 1 measurement to *D* + 1 using a 10% cutoff value [22]. Diaphragm thickening evolution was calculated as the difference in TF other the two possible intervals (*D* − 1 to SBT and SBT to *D* + 1) [29].

Low diaphragm thickening was defined as a TF below 20% [15, 16, 20, 28]. A TF below 30% is considered as a marker of potential extubation failure [19]. On *D* − 1 and on *D* + 1, US measurements were carried out during quiet breathing in a semi-recumbent position [20].

Measurements were conducted by trained investigators (first two authors), second authors had to perform at least five full exams before first inclusion under supervision of a trained physician in diaphragm ultrasound (PHM).

The primary endpoint was to assess the diaphragm thickening (TF) after elective cardiac surgery during SBT. Secondary endpoints were to assess: (1) the incidence of low diaphragm thickening (TF < 20%), (2) the time course of diaphragm thickening and thickness on *D* − 1 (“healthy conditions”) and *D* + 1 (“recovery”).

### Statistical analysis

Statistical analysis was performed using MedCalc^®^ software (Ostend, Belgium; version 11.1) under the supervision of the Grenoble Clinical Investigation Center.

Categorical variables were expressed as numbers (%), and if assumption for Chi-squared test was not fulfilled, Fisher exact test was applied. Continuous variables were expressed as mean (and standard deviation) when normally distributed or median (and interquartile [25–75%]) elsewhere and compared using Student’s *t* test or the Wilcoxon test as appropriate. Kolmogorov–Smirnov test was used to test normality.

We designed this study to assess diaphragm thickening during SBT (primary outcome) as an exploratory study. To calculate a sample size, we anticipated a 30% incidence of low diaphragm thickening (TF < 20%) according to a previously published study in difficult to wean patient [10]. This choice was pragmatic as we designed this study to describe the mechanism of difficult weaning at an early phase, using a mean of diaphragm evaluation related to the transdiaphragmatic pressure measurement and in a stress condition linked to diaphragm function. We powered this study to detect the same incidence in elective patient. To detect an effect size of 30%, incidence with 80% power and a two-tailed alpha of 0.05 required 100 participants.

Diaphragm TF was analyzed as a continuous variable. Analysis of variance (ANOVA) for repeated measures was used to compare continuous variable over time. Diaphragm TF was used to define a category of patient with TF was below 20% [15].

Pearson correlation coefficient was calculated when variables were normally distributed and Spearman rank correlation elsewhere. Inter-rater agreement was tested by Bland and Altman graphs between two measurements of one sample of ultrasound and two different investigators. Measurements were conducted independently through computer-driven software on images extracted from the data base. A *P* value of less than 0.05 was considered statistically significant.

## Results

From May to November 2014, 301 patients underwent cardiac bypass surgery. Sixty-seven patients underwent emergent procedures and 134 could not be included because of late arrival and the absence of an available trained investigator. The 100 patients included had diaphragm thickening assessment during SBT in the ICU. In 72 and 66 patients, diaphragm thickening assessment was performed twice (*D* − 1 and SBT), and three times (*D* − 1, SBT and *D* + 1), respectively. Missing data at *D* − 1 and D + 1 were explained by the absence of trained investigators.

### Patient characteristics and diaphragm thickening

Population characteristics are presented in Table 1. All patients had elective cardiac surgery with CPB and none had a history of neuromuscular disease. One patient presented sepsis (endocarditis).

**Table 1:** Population characteristics before and during surgery, according to diaphragm thickening during spontaneous breathing trial

Variables	All patients (*n* = 100)	Low diaphragm thickening during SBT (*n* = 75)	No low diaphragm thickening during SBT (*n* = 25)	*P* value
Characteristics				
Age	69 (11)	69 (10)	72 (11)	0.04
BMI (kg/m^2^)	26 (5)	27 (5)	24 (6)]	0.02
Sex (female)	26 (26)	15 (20)	11 (44)	0.1
COPD	13 (13)	11 (15)	2 (8)	0.7
Diabetes	7 (7)	6 (8)	1 (4)	1
Cancer	19 (19)	13 (17)	1 (4)	0.2
Smokers	41 (41)	32 (43)	9 (36)	0.9
Euroscore 2	1.6 [1–3]	1.6 [1–3]	1.6 [1–3]	0.7
Preoperative LVEF (%)	59 (12)	58 (12)	63 (8)	0.3
Sepsis	1 (1)	1 (1)	0 (0)	1
Type of surgery				
CABG	46 (46)	37 (49)	9 (36)	0.6
Isolated	37 (37)	29 (39)	8 (32)	0.8
Grafts				
Left mammary artery	45 (45)	36 (48)	9 (36)	0.7
Right mammary artery	27 (27)	23 (31)	4 (16)	0.3
Gastroepiploic artery	11 (11)	10 (13)	1 (4)	0.5
Saphenous vein	14 (14)	13 (17)	1 (4)	0.2
Radial artery	4 (4)	2 (3)	2 (8)	0.3
Valvular surgery	59 (59)	44 (59)	15 (60)	0.9
Isolated	37 (37)	25 (33)	12 (48)	0.5
Aortic	47 (47)	35 (47)	12 (48)	0.8
Mitral	12 (12)	9 (12)	3 (12)	1
Pulmonary	3 (3)	3 (4)	0 (0)	1
Ascending aorta	16 (16)	13 (17)	3 (12)	0.8
Combined surgery	24 (24)	20 (27)	4 (16)	0.6
Thoracotomy	6 (6)	5 (7)	1 (4)	1
Other	3 (3)	1 (1)	2 (8)	0.2
Cooling				
Cool gauze	65 (65)	50 (67)	15 (60)	0.9
Ice saline	18 (18)	12(16)	6 (26)	0.7
Pericardial ice	11 (11)	7 (9)	4 (16)	0.5
No cooling	6 (6)	6 (8)	0 (0)	0.3
CPB time (min)	97 (33)	103 (35)	83 (24)	< 0.01
Cross-clamp time (min)	59 [47–73]	61 [48–82]	51 [44–59]	0.01
Surgery duration (h)	5.5 (1)	5.7 (1)	4.8 (0.7)	< 0.01
Postoperative MV (h)	3.4 [3, 4]	3.3 [3, 4]	3.6 [3, 4]	0.08
Total MV (h)	9 [8–10]	9.1 [8–10]	8.6 [8–10]	0.5
Vasopressor	32 (32)	24 (32)	8 (32)	0.8
Propofol (mg)	1326 (439)	1388 (455)	1159 (351)	< 0.01
Remifentanil (µg)	2674 (812)	2873 (763)	2100 (694)	< 0.01
NMBA (mg)	20 [20–28]	24 [20–30]	20 [17–20]	0.06

A low diaphragm thickening was defined as a thickening fraction < 20%

Data are presented as mean and standard deviation, median with first and third quartile or number [−] and percentages ()

*BMI* body mass index, *COPD* chronic obstructive pulmonary disease, *LVEF* left ventricle ejection fraction, *CPB* cardiopulmonary bypass, *MV* mechanical ventilation, *NMBA* neuromuscular blocking agent

The cardiac surgery distribution reflects the usual activity of a cardiac surgery centers, i.e., evenly balanced between coronary bypass and aortic valve surgery (Table 1). The breathing pattern, hemodynamics and oxygenation parameters are presented in Table 2. The PaO2/FiO2 ratio was higher when diaphragm thickening was above the TF value of 20% (*P* = 0.02).

**Table 2:** Physiological measurements, according to diaphragm thickening during spontaneous breathing trial (SBT) with the settings used during the ultrasound assessment (pressure support and positive end-expiratory pressure set at 0 cmH_2_O)

Variables	All (*n* = 100)	Low diaphragm thickening during SBT (*n* = 75)	No low diaphragm thickening during SBT (*n* = 25)	*P* value
Physiological data				
TV (ml)	426 (120)	439 (125)	395 (99)	0.1
RR (cycles/min)	24 [20–27]	23 [20–28]	22 [20–26]	0.6
RR/TV	62 (29)	61(28)	63 (27)	0.5
SpO2 (%)	100 [98–100]	100 [98–100]	100 [98–100]	0.3
Mean arterial pressure (mmHg)	75 (18)	75 (16)	78 (14)	0.9
Heart rate (bpm)	78 (14)	80 (12)	75 (18)	0.2
PaO2/FiO2	303 (75)	302 (66)	341 (89)	0.02
PaCO2 (mmHg)	37 (6)	38 (6)	36 (5)	0.1

Data are presented as mean and standard deviation, median with first and third quartile or number [–] and percentages ()

*TV* tidal volume, *RR* respiratory rate, *SBT* spontaneous breathing trial, *bpm* beats per minute, *TF* thickening fraction

**Fig. 2:**
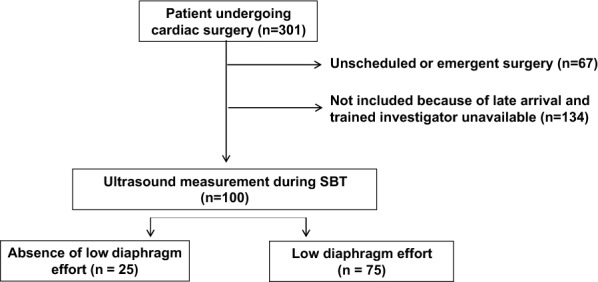
Study flow chart

Ultrasound measurement during SBT found a mean *T*_EE_ of 2.3 mm (± 0.6) and a mean thickness at end inspiration (*T*_Ei_) of 2.6 mm (± 0.7). Mean overall diaphragm thickening was 17% (± 14). Low TF incidence was 75% (Fig. 2) during SBT and 84% patients had a TF < 30% during SBT. At *D* − 1, the incidence of a TF below 20% was 15% (11 patients). We performed a comparative analysis of the data obtained from the US measurements of the left and the right hemidiaphragm during SBT. These data are provided separately as online supplementary material (see Additional file 1 and Additional file 2: Fig. 1).

### Outcome

Two patients required reintubation, one because of an emergency surgery for postoperative pericardial effusion and one because of an acute respiratory failure secondary to postoperative multiple organ failure (one patient). Length of total mechanical ventilation and postoperative mechanical ventilation duration was not longer in case of low diaphragm thickening.

### Time course of diaphragm characteristics

In patients assessed during the three time points (*n* = 66), mean *T*_EE_ did not change significantly 2.4 mm (± 0.5) during preoperative assessment, 2.3 mm (± 0.6) during SBT and 2.4 mm (± 0.5) on *D* + 1. Preoperatively, 34% of the patients had a *T*_EE_ of less than 2 mm and 0 had a *T*_EE_ below 1.5 mm [15].

**Fig. 3:**
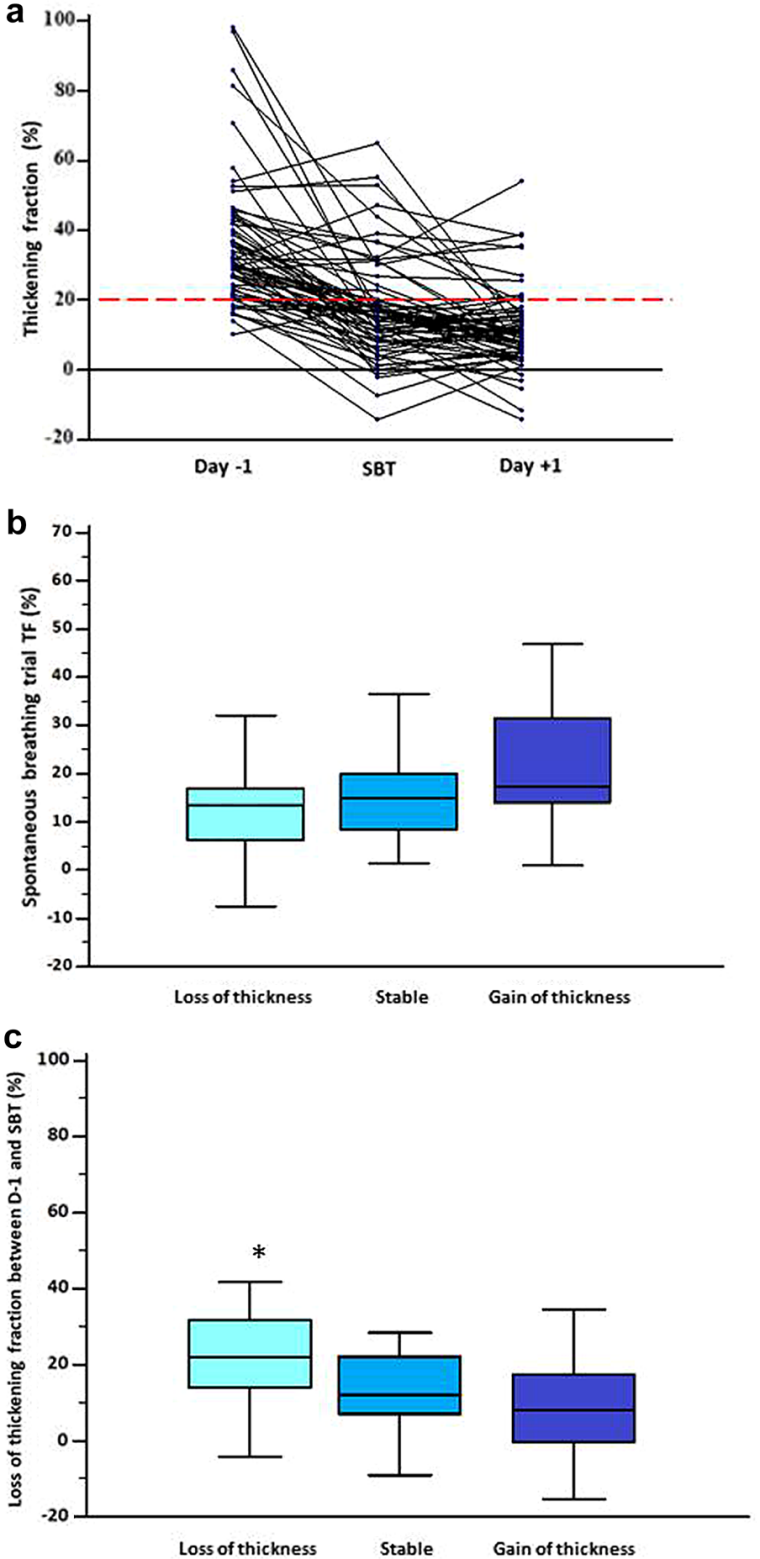
Evolution of diaphragm thickening fraction and its impact on thickness. **a** Individual values of perioperative ultrasound measurement of diaphragm thickening. Diaphragm function was assessed by calculation of right diaphragm thickening fraction (TF). A TF below 20% during SBT (black dashed line) defined low diaphragm thickening during. In this study, 66 patients had three measurements. The first assessment was made the day before surgery 36% (± 18), the second assessment was made at the time of spontaneous breathing trial (SBT) 17% (± 14) and the third, on day one postoperatively 12% (± 11). Repeated analysis of variance showed a significant difference between groups (*P* < 0.0001). **b** TF during SBT according to the three different thickness patterns. First column named loss of thickness was defined as a decrease of > 10% from baseline, second column named stable was defined by the absence of changes above 10% from baseline and third column named gain of thickness was defined by an increase of > 10% from baseline. The TF was in case of loss of thickness (14% (± 13)), versus stable patients (16% (± 14) and gain of thickness patients (23 (± 16)), ANOVA one-way analysis of variance *P *= 0.1. **c** Evolution of diaphragm thickening between preoperative day (*D* − 1) and SBT according to the three different thickness patterns. First column named loss of thickness was defined as a decrease of > 10% from baseline, second column named stable was defined by the absence of changes above 10% from baseline and third column named gain of thickness was defined by an increase of > 10% from baseline. The diaphragm thickening had a higher decrease in case of thickness loss (25% (± 17)), versus stable patients (15% (± 15) and gain of thickness patients (11 (± 21)), ANOVA one-way analysis of variance *P *= 0.02. *P < 0.05. TF: thickening fraction; SBT: spontaneous breathing trial

In the 66 patients assessed during the three time points, diaphragm thickening was significantly lower during SBT and *D* + 1 compared to baseline (Fig. 3a) (36% (± 18) vs 17% (± 14) vs 12% (± 11), respectively; *P* < 0.0001, ANOVA). The TF during SBT was correlated to the *D* + 1 TF (*r* = 0.46 [0.24–0.63]; *P* < 0.0001).

**Fig. 4:**
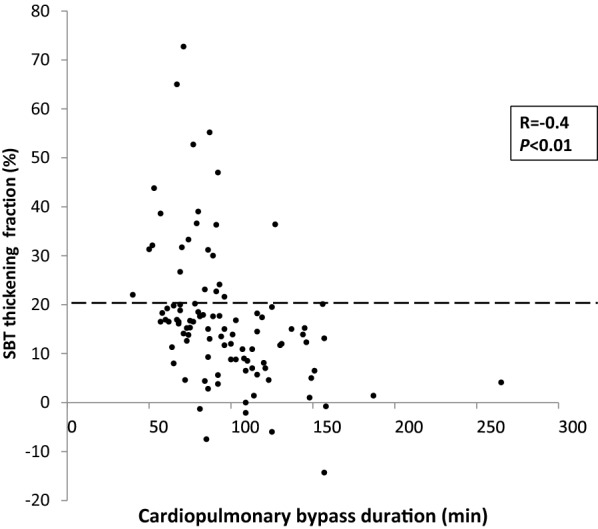
Individual values of diaphragm thickening fraction according to cardiopulmonary bypass duration. The diaphragm thickening fraction (TF) during the spontaneous breathing trial (SBT) was recorded according to the Y, and the cross-clamp time was recorded according to the X. Diaphragm function was reduced when cardiopulmonary bypass time was longer (*r* = − 0.4; *P* < 0.0001). SBT: spontaneous breathing trial; TF: thickening fraction

Univariable analysis for demographic and clinical characteristics is presented in Table 1. At SBT, diaphragm thickening was not related to the type of surgery or the cooling methods (Table 1). Duration of cross-clamping, CPB and surgery was prolonged in patients with low TF, and they were correlated to TF at SBT (Fig. 4): duration of cross-clamping (*r* = − 0.3; *P* = 0.0007), CPB (*r* = − 0.4; *P* < 0.0001) and surgery length (*r* = − 0.4; *P* < 0.0001). Along with the procedure duration, total intraoperative remifentanil and propofol were higher in case of low diaphragm thickening (Table 2) but not NMBA. Remifentanil and propofol were correlated to TF at SBT: *r* = − 0.47; *P *< 0.0001 and *r* = − 0.4; *P *< 0.0001).

From baseline during the preoperative day to the postoperative day one, 19 patients (29%) *T*_EE_ increased, 19 patients (29%) as well had a stable *T*_EE_ and for 28 (42%) *T*_EE_ decreased. Length of stay was higher in case of decrease, ANOVA (*P *= 0.046). The percentage of changes in thickness is related to the length of stay (*r* = − 0.281, *P *= 0.0234). Measurement of diaphragm thickening during SBT was not different according to the thickness evolution (Fig. 3b). Loss of TF between *D* − 1 to SBT was higher in the decrease group regarding the two others, ANOVA (*P *= 0.02), (Fig. 3c and Table 3).

**Table 3:** Demographic, preoperative and diaphragm variables associated to the change in diaphragm thickness

	Change in diaphragm thickness between day one preoperatively and day + 1 after surgery			
	> 10% of decrease	Stability	> 10% of increase	
	*n* = 29	*n* = 19	*n* = 19	*P* value
Age (yo)	73 [66–81]	66 [59–74]	74 [58–76]	0.2
BMI (kg/m^2^)	26 (4)	27 (4)	25 (4)	0.2
Euroscore 2	2 [1–4]	1 [1–3]	1 [1, 2]	0.3
Length of stay (d)	3 [2–5]	2 [2–4]	2 [2]	0.046
RR (cycles/min)	25 (8)	22 (7)	24 (8)	0.3
TV (ml)	411 (135)	432 (123)	436 (124)	0.8
RR/TV	67 (30)	62 (28)	58 (26)	0.6
Cross clamp time (min)	62 (24)	74 (35)	61 (35)	0.4
Surgery duration (h)	5.4 (1)	5.8 (1)	5.2 (1)	0.2
Total MV (h)	10 (4)	9 (1)	9 (2)	0.2
CPB time (min)	93 (25)	103 (35)	90 (48)	0.5
Postoperative MV (h)	4.5 (3)	3 (1)	3.3 (1)	0.1
*T*_EE_ preoperative (mm)	2.6 (0.6)	2.4 (0.4)	2.1 (0.4)	0.002
*T*_EE_ during SBT (mm)	2.4 (0.7)	2.2 (0.5)	2.2 (0.6)	0.5
*T*_EE_ day one (mm)	2 (0.4)	2.3 (0.4)	2.7 (0.6)	< 0.001
Preoperative TF (%)	40 (20)	30 (14)	34 (18)	0.7
SBT TF (%)	14 (13)	16 (14)	23 (16)	0.1
Day one TF (%)	13 (12)	9 (8)	12 (13)	0.6
Loss of TF from *D* − 1 and SBT	25 (17)	15 (15)	11 (21)	0.02
Loss of TF from SBT to *D* + 1	− 2 (14)	− 6 (12)	− 11 (14)	0.08
Loss of tf from *d* − 1 to *d* + 1	27 (20)	21 (12)	22 (21)	0.5

Data are presented as mean and standard deviation, median with first and third quartile or number [–] and percentages ()

*BMI* body mass index, *TV* tidal volume, *RR* respiratory rate, *SBT* spontaneous breathing trial, *T*_*EE*_ thickness at end expiration, *TF* thickening fraction TV, *CPB* Cardiopulmonary bypass, *MV* mechanical ventilation

### Inter-rater variability for US measurement

Inter-rater agreement was tested by the Kappa test on a sample of 14 patients assessed for *T*_EE_ and *T*_EI_ (28 means of three measurements) by two different investigators. Kappa was 0.959 ± 0.267 when the TF was calculated for each observer, standard deviation was 4% with a limit of agreement (− 8% and 7%). A Bland–Altman graph is presented as a supplemental material (see Additional file 3: Fig. 2).

## Discussion

The findings of this study indicate that (1) diaphragm thickening can be markedly reduced in the postoperative cardiac surgery with an incidence of 75% of patients assessed with a TF below 20%; (2) the time course of diaphragm thickening was associated with diaphragm thickness changes.

Diaphragm TF is usually altered postoperatively. The incidence of 75% found here is higher than in other studies where rates between 1.2 and 60% were reported [6]. Diaphragm ultrasound assessment may play a key role to study diaphragm weakness. A recent article pointed out the correlation between diaphragm function assessed by magnetic stimulation and TF during tidal breathing [21]. The 20% cutoff was initially described to define diaphragm dysfunction [14]. The low TF values observed in the current study may reflect that diaphragm thickening was low because of mildly affected respiratory mechanics after cardiac surgery unlike other ICU patients. None of our patient experienced high catabolic state imposed by septic shock or neuromuscular impairment linked to multiple organ failure. This assumption might be accepted as diaphragm thickening is related to the diaphragm effort. However, accounted that measurement were made under a stress conditions, therefore reflecting diaphragm function as well, the results relevantly implies an impaired diaphragm strength as well. Whether these acquired characteristics are transient or recover would need a longer follow-up. However, this study provides an exploration of inspiratory mechanics in the settings of elective cardiac surgery. This case mix may explain the low weaning failure rate. However, we found an interesting association between thickness evolution and ICU stay duration.

Diaphragm characteristics have been investigated during ventilation and weaning. In vitro data report impaired contractility after 2 h of mechanical ventilation [30]. In the present study, duration of surgical procedure was associated with diaphragm thickening loss. This can be explained by phrenic nerve trauma produced by cooling devices or direct surgical lesions [31].

This study reports for the first time to our knowledge that diaphragm thickening is related to thickness evolution in this particular population and condition of cardiac surgery. We studied three diaphragm thickness evolution patterns, and we interestingly found that patient who developed loss of thickness experienced an extended length of stay in the ICU [26]. The moderate outcome impairment might be explained by our case mix of elective patient. Further studies including emergent or salvage surgery patient would be of interest to confirm this trend. These findings are consistent with previous studies as changes of thickness within the first day of respiratory failure were related with diaphragm thickening [13, 22]. However, our results showed a high incidence of increased thickness (29%) in an apparently short time frame. It may be explained firstly by the fact that baseline measurement is a healthy comparator because patients were enrolled before any mechanical ventilation, anesthesia and surgery procedure. Secondly, the 2 days between the first and the final assessment necessary to produce this thickness changes, which can be considered as a short duration, are a comparable time frame with the results found in other publications [22]. Muscle impairment probably does not stop with the surgery and the postoperative mechanical ventilation. The postoperative day one TF measurements were lower than the previous measurements which could be interpreted as an ongoing contractile impairment. These results support recently reported evidences that contractile activity influences the diaphragm thickness and impacts patient outcome [29]. Along diaphragm effort impairment, given the close link between diaphragm function and the muscle contractile activity, the hypothesis that diaphragm dysfunction promotes thickness changes remains relevant.

Our results show a relation between intraoperative sedative agents and the diaphragm thickening during the SBT. Evidences of an impact of sedatives on diaphragm function and respiratory pattern exist [32, 33]. However, the results of the present study are difficult to isolate from the surgery duration and mechanical ventilation as drug doses reported were only intraoperative doses, and we do not report the ICU doses. Further studies including different sedatives strategies should investigate the impact on respiratory muscle to investigate the hypothesis of a direct effect.

Diaphragm ultrasound has some pitfalls. The relation between diaphragm thickness and its predictor variable TF index needs cautions. We should consider that changes in thickness like an acquired significant atrophy or edema may involve modification in TF calculation. Systematic small changes in thickness measurements might lead to false conclusions. However, the incidence of changes in diaphragm thickness is consistent with recent studies published in the context of ICU patients [26]. Therefore, along with others, we may hypothesis that thickness changes could equally affect inspiration and expiration [21].

The major strength of this study is the inclusion of a large number of patients after unselected elective cardiac surgery to describe the specific diaphragm thickening and myotrauma pattern. The ultrasonic technique used for the measurement of diaphragm thickening during tidal breathing is non-volitional and was applied during the spontaneous breathing trial. Our study presents some limitations. First, we did not use magnetic stimulation of the phrenic nerve which is the gold standard to assess diaphragm dysfunction [34]. Since patients may require a pacemaker during the postoperative period, using this method could be harmful [8]. Second, measurements during quiet breathing were compared between intubated and extubated patients, which may result in different diaphragmatic loads. To reduce this bias, patients were assessed with a PSV of 0 cmH_2_O and a PEEP level of 0 cmH_2_O which was described in other studies and also recognized to accurately reproduce the post-extubation conditions [25, 35]. Third, although the standardization of probe placement was respected throughout the study, we did not use markers to maximize reproducibility between exams for the same patient [36]. Fourth, we studied patient exposed to different cooling methods, which could be a cofounding factor, even though we did not find significant differences. Fifth, we focused the respiratory complication on reintubation. Nevertheless, considering its low incidence in elective surgery, it may not be relevant as it does not reflect the diversity of respiratory complications and their impact on patient’s recovery [37]. Sixth, we did not include patient in the absence of trained investigator resulting in loss of data on longitudinal follow-up. Therefore, it did not allow the use of adjusted statistics on this secondary outcome. Our choice was to show these results as we did not previously planned replacement method for missing secondary outcome, and power calculation was not made on these exploratory variables. We defined a trained investigator as an investigator with at least five full sessions which is a relatively small number of exams but considered as sufficient for some authors [19].

## Conclusion

We reported a prospective study involving 100 patients undergoing elective cardiac surgery. We found a reduced diaphragm thickening during SBT and on the first postoperative day compared to the preoperative day. Diaphragm thickness was influenced by diaphragm thickening, and its evolution was associated with a longer length of stay. Although diaphragm thickening was not associated with extubation failure, it may influence diaphragm thickness and patient outcome. Further studies would need to focus on longer follow-up, including the diversity of respiratory complications and higher risk patients.

## Additional files

**Additional file 1.** Left diaphragm analysis.

**Additional file 2.** Individual values of the right hemi-diaphragm thickening fraction according to the left hemi-diaphragm thickening fraction. The right hemi-diaphragm thickening fraction (TF) during the spontaneous breathing trial (SBT) was reported according to the Y and left hemi-diaphragm thickening fraction (TF) during the SBT according to the X (*r* = 0.57 [0.4–0.7]: *P* < 0.001).

**Additional file 3.** Comparisons of right hemi-diaphragm thickness assessed independently by two different raters during a spontaneous breathing trial.

## Abbreviation

ANOVA: analysis of variance
CECIC: Comité d’éthique des centres d’investigations cliniques
CPB: cardiopulmonary bypass
*D* − 1: day before surgery
*D* + 1: day after surgery
FiO2: inspired fraction of oxygen
ICU: intensive care unit
LOS: length of stay
PEEP: positive end-expiratory pressure
PSV: pressure support ventilation
SBT: spontaneous breathing trial
*T*_EE_: diaphragm thickness at end of expiration
*T*_EI_: diaphragm thickness at end of inspiration
TF: diaphragm thickening fraction
