# Supplementary material for: Diaphragm thickening in cardiac surgery: a perioperative prospective ultrasound study
Source: Ann Intensive Care. 2019 Apr 24;9:50. doi: 10.1186/s13613-019-0521-z (PMC6478777; doi:10.1186/s13613-019-0521-z)
Supplement: Supplementary file 1 — Additional file 1. Left diaphragm analysis. [file 13613_2019_521_MOESM1_ESM.docx]

**Additional file 1**

**Left diaphragm analysis**

**Material and methods**

Left-hemidiaphragm measurement were completed during SBT with the same settings as for the right diaphragm. Bland-Altman graphs were used to compare right and left-hemidiaphragm.

**Results**

We performed a comparative analysis of the data obtained from the US measurements of the left and the right hemidiaphragm during SBT. This was possible in only 69 patients owing to a poor ultrasound window on the left hemi-diaphragm. Left and right TF were not different statistically (19 % and 17% (± 14), *P*=0.33) and correlated among this population (r = 0.57 [0.4-0.7]: *P* < 0.001, supplemental figure2).

**Discussion**

Comparison between the right and left hemi-diaphragm thickening present an acceptable correlation for the interpretation of the overall diaphragm mechanics. Despite a higher incidence of left mammary artery graft, the left TF was not lower. It questions the role of the surgical trauma in diaphragm palsy and the threshold of diaphragm thickening fraction of the left hemi-diaphragm.

# Additional file 2: Individual values of the right hemi-diaphragm thickening fraction according to the left hemi-diaphragm thickening fraction.

The right hemi-diaphragm thickening fraction (TF) during the spontaneous breathing trial (SBT) was reported according to the Y and left hemi-diaphragm thickening fraction (TF) during the SBT according to the X (r = 0.57 [0.4-0.7]: *P* < 0.001).

**Additional file 3: Bland-Altman plot.**

Comparisons of right hemi-diaphragm thickness assessed independently by two different raters during a spontaneous breathing trial.
