# Supplementary figures and images for: Diaphragm thickening in cardiac surgery: a perioperative prospective ultrasound study
Source: Ann Intensive Care. 2019 Apr 24;9:50. doi: 10.1186/s13613-019-0521-z (PMC6478777; doi:10.1186/s13613-019-0521-z)

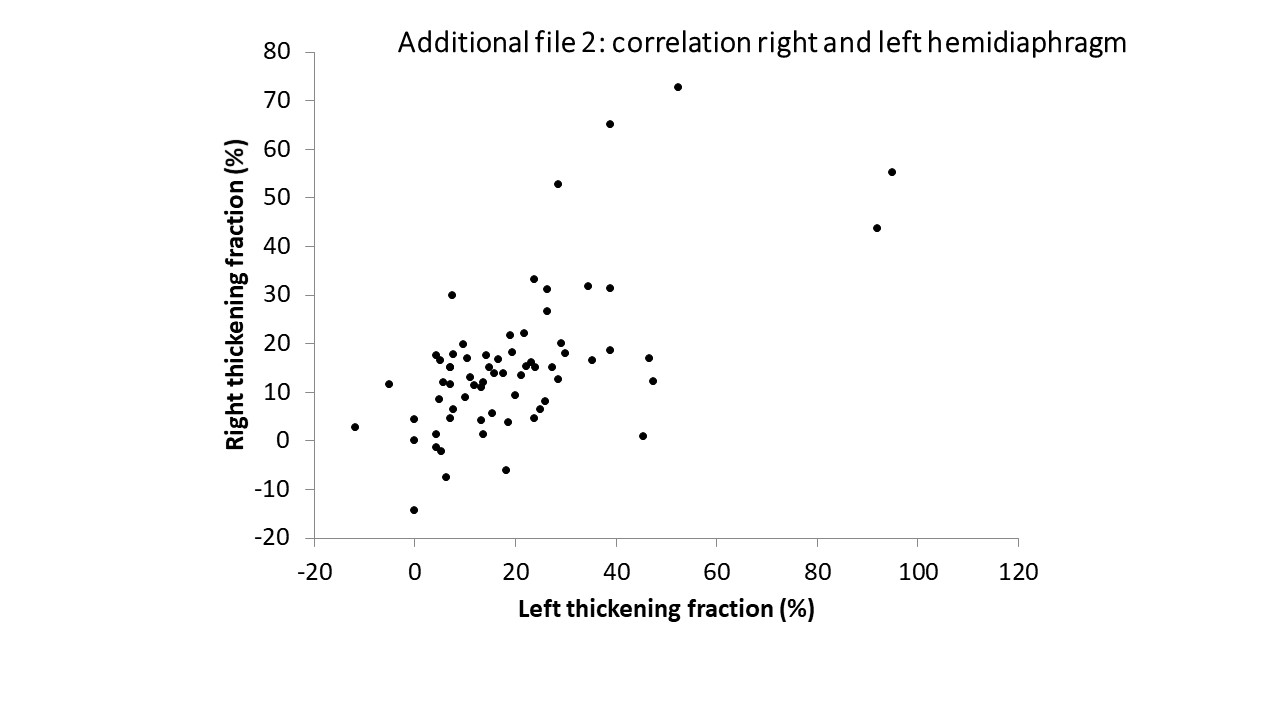

Supplement: Supplementary file 2 — Additional file 2. Individual values of the right hemi-diaphragm thickening fraction according to the left hemi-diaphragm thickening fraction. The right hemi-diaphragm thickening fraction (TF) during the spontaneous breathing trial (SBT) was reported according to the Y and left hemi-diaphragm thickening fraction (TF) during the SBT according to the X (r = 0.57 [0.4–0.7]: P < 0.001). [file 13613_2019_521_MOESM2_ESM.jpg]

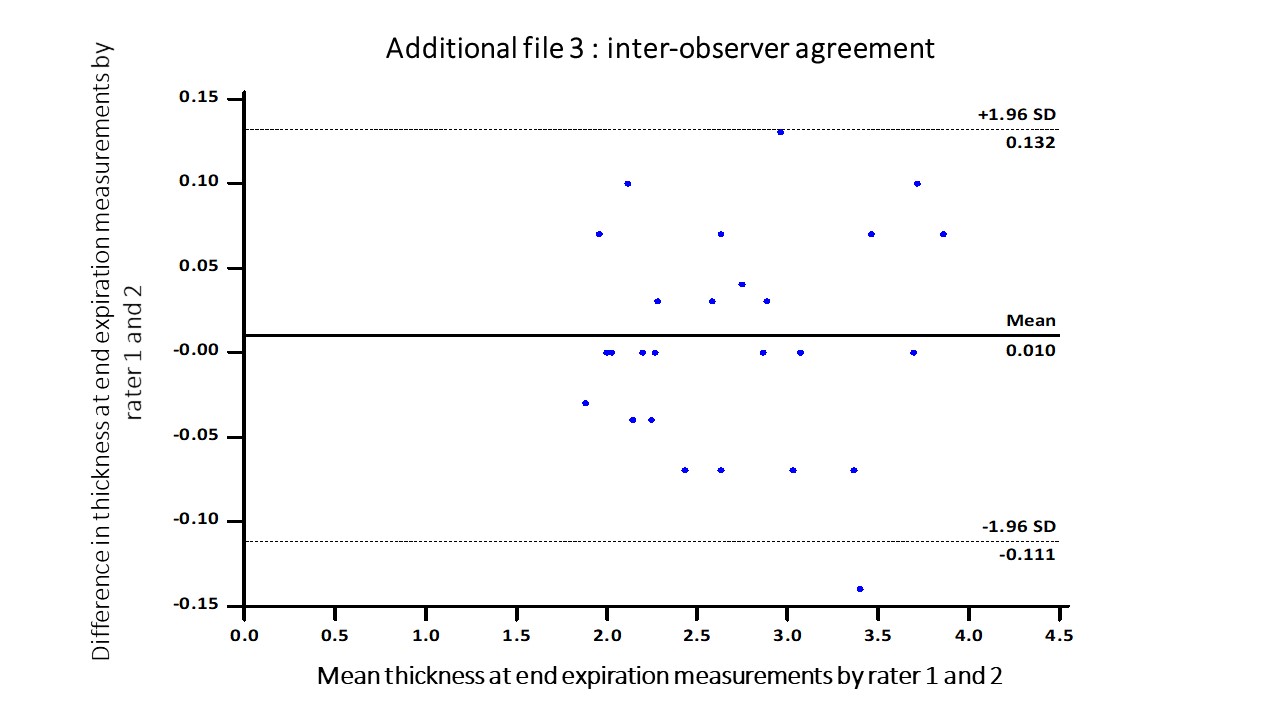

Supplement: Supplementary file 3 — Additional file 3. Comparisons of right hemi-diaphragm thickness assessed independently by two different raters during a spontaneous breathing trial. [file 13613_2019_521_MOESM3_ESM.jpg]
